# Changing the Story on Health and Racial Equity: Why Public Health Needs an Infrastructure for Building Narrative Power

**DOI:** 10.1111/1468-0009.70047

**Published:** 2025-08-20

**Authors:** LORI DORFMAN, SARAH E. GOLLUST, MAKANI THEMBA, PRITPAL S. TAMBER, ANTHONY ITON

**Affiliations:** ^1^ Berkeley Media Studies Group Public Health Institute; ^2^ University of California Berkeley School of Public Health University of California Berkeley; ^3^ University of Minnesota School of Public Health University of Minnesota Twin Cities; ^4^ Higher Ground Change Strategies; ^5^ Pritpal S Tamber Consultorio Ltda; ^6^ The Health Trust

**Keywords:** community organizing, narrative, communication, power, health equity

## Abstract

**Policy Points**

- One form of power that is required for advancing health and racial equity is narrative power: the ability to shift the stories we use to make sense of the world.
- Building this form of power requires the field of public health to strategically work to connect institutions and organizations to align in complementary ways to create, build, and sustain new narratives—what we refer to as narrative infrastructure.
- We illustrate these ideas using real‐world examples drawn from work in tobacco control and emerging work in addressing structural racism in public health.

A growing body of scholarship and practice in public health attests to the importance of addressing differences in power as a fundamental determinant of health inequities.^1^, ^2^, ^3^, ^4^, ^5^, ^6^ To pursue health equity, public health practitioners must move beyond identifying differences in health outcomes among populations (disparities) to articulating why those differences are unfair or unjust (inequities) and then identifying structures, such as laws, policies, practices, and norms, that advantage some and disadvantage others.^7^, ^8^ The act of doing so forces public health practitioners to consider the allocation of public resources, a process that is ultimately political.^3^, ^9^ Fundamentally, going this far “upstream” to the root causes of inequity means confronting, understanding, and then addressing differences in power among and across populations.^2^, ^10^

Within the context of public health, Iton and colleagues^3^ contend that advancing health and racial equity requires building *community power*, which they define as “the ability of people facing similar circumstances to develop, sustain, and grow an organized base of people who act together through democratic structures to set agendas, shift public discourse, [and] influence who makes decisions.”^3^ ^p1764^ Building community power is typically done through community organizing, recently defined for a health audience as “the processes by which people who have a common identity or purpose unite to build relationships, identify shared issues, collectively analyze those issues to understand structural injustices, develop collective goals based on that analysis, and implement strategies and tactics to reach those goals.”^11^

A number of frameworks have emerged from the practice and theory of community organizing that identify ways to form, build, and wield power.^12^, ^13^, ^14^, ^15^, ^16^ Crucially, much of the knowledge about building community power was (and continues to be) created by “frontline” communities most impacted by inequity. This lineage of community‐based knowledge from indigenous communities and others is extensive and evolving and includes the work of many, mostly unnamed and unattributed thinkers and strategists from across the globe—many of whom are people of color. Although their work and contributions are not (yet) cited often in the academic canon, their influence on our understanding of power and inequity is substantial. This includes the work and writings of civil and human rights leaders like Dr. Martin Luther King Jr, who wrote that power is “the ability to achieve purpose. It is the strength required to bring about social, political, or economic changes.”^17^ Other influential organizers whose work continues to shape power‐building theory and practice include Dolores Huerta, Cesar Chavez, James and Grace Lee Boggs, Clyde Bellecourt, Dennis Banks, Fannie Lou Hamer, and Jane McAlevey, among others (to learn more about their work, see, e.g., Boggs and Kurashige,^18^ Sowards,^19^ Ransby,^20^ and Banks^21^).

Many of the tactics identified in community organizing frameworks implicitly or explicitly involve broadly defined communication. This includes articulating clear goals, talking with allies to build a base of people and coalitions willing to act, or using the media in advocacy campaigns (see Appendix Table 1 for a display of common elements of three existing power‐building frameworks in organizing practice). Broader still, building power also requires shifting the public's values and beliefs about how the world works through shifting widely shared, overarching *narratives*. The act of doing so requires a set of activities, including building capacities, establishing relationships, and cultivating networks, which have come to be known as building *narrative infrastructure*.^22^, ^23^, ^24^

In this perspective, we define the multiple and intersecting roles of narrative and narrative infrastructure in power building, clarifying these terms for public health research, practice, and teaching audiences. We then argue that creating an infrastructure to build narrative power is essential to bring about the structural changes needed to pursue health and racial equity. We illustrate these ideas using real‐world examples drawn from work in tobacco control and emerging work in addressing systemic racism and provide specific tactics that public health researchers, practitioners, and their multisector allies and partners can leverage to build narrative power.

## Defining Terms and Bridging Fields: Narratives for Health and Power Building

The construct of narrative is used in different ways across different fields. In contexts of health communication, such as communication campaigns aiming to shift an audience's beliefs or actions, the construct of narrative refers to how health messages can be packaged into stories. For instance, a health campaign might be designed around a story of a protagonist facing a health struggle who overcomes that problem through the successful receipt of a health service.^25^ These health communication narratives are often oriented around individuals, but practitioners also use narrative formats to present information about the broader social or systemic context surrounding health and social issues.^26^ For example, media advocacy, the strategic use of media to support community organizing and advance public policy, emphasizes the use of stories that clearly locate problems within social and structural factors and identifies specific policy solutions so as to direct attention among the public and policymakers to these solutions.^27^, ^28^, ^29^

In community organizing contexts, the construct of narrative means something a bit different. Narrative in this context is not (just) the packaging of information into the format of a story about individuals or the strategic repetition of messages about particular policy solutions. For organizers, narratives are how we all make sense of the world. Narratives are the aggregation of stories that convey values and worldviews, thus shaping meaning at scale.^4^, ^30^ When these stories (and their underlying values and worldviews) are shared by many of us, they become *dominant narratives* that permeate our mindsets, constrain how we think about health and other issues, and shape our understanding of the types of actions or solutions that could transform unhealthy conditions. For instance, one dominant narrative within the United States is that of rugged individualism: the idea that we are all individually responsible for our health and health behaviors, emphasizing the value of personal responsibility.^31^ Another dominant narrative is the concept of deservingness: the idea that we get what we deserve based on the work we put in, so poor outcomes result from a lack of personal effort.^32^

These dominant narratives have concrete effects on the ways we collectively think about health problems and their solutions. One specific health policy example is the ingrained connection between employment and deservingness of health insurance. Employer‐based health insurance is the primary mechanism through which people below 65 years of age in the United States access health insurance.^33^ This link—created through historical policy and sustained in public understanding of what it means to deserve health insurance—contributes to the worldview that leads people to unquestioningly consider a new job, or the decision to stay in a current job, based on the health insurance benefits that job provides. At the same time, the narrative casts those who are unemployed as undeserving of insurance. This dominant narrative of health insurance deservingness through employment also contributes to the political reality and even political viability of certain policy proposals, such as those that make Medicaid coverage contingent on employment. Furthermore, this dominant narrative means that policies that aim to unlink health insurance status from employment are viewed as politically improbable. It also reinforces the worldview that individual responsibility and hard work determine who is deserving of health care, which constrains the political possibilities for changing health insurance policy.

The existence of these dominant narratives is neither an accident nor a coincidence. They reinforce systems that benefit those who promulgate them: in this instance, health insurance companies and others who profit from privatized health care in the United States. As Jonathan Heller, a public health leader who (alongside colleagues) has helped bring community organizing concepts into the practice of public health, articulates, “The ability to influence which narratives are dominant is a form of *power*—the narratives that are dominant shape what is thought of as commonsense in our society, and therefore make some policy proposals seem like no‐brainers and others feel like they were beamed in by aliens.”^34^ The narrative linking work and health makes employer‐based health insurance seem natural and apolitical, the “no‐brainer,” which means that alternatives to employer‐based health insurance, such as all people having a right to health care, seem “alien,” or radical political ideas. Entities or people have *narrative power* when they have the capacity to shape the narratives that become dominant or are treated like common sense.

## Narrative Power and Health Equity

We define narrative power as “the ability to make the foundational stories we tell—the stories about how things work, our sense of history, who and what matters, and our relationship to one another and the planet—the main stories people use to make sense of the world over time.”^35^ ^p7^ Narrative power shapes “people's conscious and unconscious understandings of the world, of what is politically possible, and of their own place in the world” through institutions with broad social reach, from mass media, religious or educational institutions, the legal system, political parties, and more.^14^ The concept of narrative power has a deep intellectual origin, drawing from the constructs of the “three faces of power” from Lukes^36^ and the idea of hegemony from Gramsci,^37^ both of whom argue that one important element of power is controlling societal narratives or widespread beliefs.

Using the example above, the dominant narrative of health insurance linked to employment is part of a set of narratives that uphold the overarching narrative of capitalism. An overarching narrative is the big story that explains the little stories.^30^ Like other narratives, overarching narratives use tropes, like pulling yourself up by your bootstraps or other storytelling conventions, which evoke characters, plot, and scenes—the characteristics that help people make sense of what they see around them. The overarching narrative of capitalism—especially America's form of capitalism in which race structures labor hierarchies—makes various other narratives about deservingness, poverty, wealth, and public priorities cohere. These narratives mutually reinforce the paired ideas that personal initiative drives success in health and other endeavors as well as the flip side: if the individual fails, it is their own fault. These dominant narratives focus our attention on individual actions and obscure the social, structural, or commercial determinants of health that shape physical conditions and favor market imperatives for profit over health.^30^, ^38^, ^39^, ^40^

As a consequence of these narratives, the public perceives higher rates of disease in populations of color as more likely attributed to personal and cultural failings rather than the consequence of policy decisions that shape the disparate conditions in which people live.^41^ Public health understandings of structural factors (and structural racism in particular) as a fundamental driver of health, then, would represent a critical counternarrative. As Williams and colleagues^42^ assert, “The persistence of racial inequities in health should be understood in the context of relatively stable racialized social structures that determine differential access to risks, opportunities, and resources that drive health.” Therefore, as they and others have argued, interventions that address structural racism are necessary to promote health equity.^42^, ^43^, ^44^ Understanding and wielding narrative power to create narratives that can take root, illuminate structural solutions, and ultimately become dominant is therefore crucial for advancing health equity. To shift narratives, public health needs to build narrative power.

Importantly, narrative power is not the same thing as identifying a *powerful narrative*, a communication message that is persuasive or resonant. Narratives do not take hold simply by repeating a message many times, regardless of how compelling or persuasive that message is. Identifying a message that can persuade people that, for instance, racism is a threat to health is not narrative power. The identification of such resonant messages is an important tactic for shifting public understanding but is insufficient, on its own, to constitute narrative power. This is because that single story is not rooted in the institutions or structures that help people make meaning over time. In contrast, narrative power is the capacity to ensure that specific stories and ideas become *the* stories and ideas that people hold in common and that are rooted in and reproduced by key institutions that make meaning, like schools, government, faith institutions, media (news, entertainment, advertising), and museums.

## Components of an Infrastructure for Building Narrative Power

Building narrative power requires building infrastructure—the institutions, actors, organizational networks, and systems of cultural production and reproduction that collectively engage and organize to establish broad yet complementary actions that, taken together, build and maintain the narratives that people use to make sense of the world (see Table 1). The infrastructure would support people as they do the hard work of dismantling and replacing the narratives that undergird racism and inequitable conditions that harm health. These current dominant narratives are not accidental, but they are purposefully propagated and sustained by existing forces that benefit from the status quo, thus requiring a strategic, organized, and powerful response.^45^ For example, anti‐equity forces have consolidated wide‐ranging narrative infrastructure across a range of systems and structures. This infrastructure includes vast media holdings like those of Amazon's chief executive officer, Jeff Bezos, who has recently leveraged his power as owner to shift the editorial leanings of the *Washington Post* toward “support and defense of…personal liberties and free markets.”^46^ Anti‐equity groups have also built narrative infrastructure among aligned faith institutions and faith oriented media, film production and distribution, school curricula and education policy, and popular music.^47^, ^48^, ^49^, ^50^, ^51^, ^52^ Consensus is growing on the importance of building narrative infrastructure among organizing and power‐building practitioners, especially in the face of these challenges.^22^, ^23^, ^24^ Furthermore, there are many existing examples of narrative work happening within public health organizations including, but not limited to, work by organizations like County Health Rankings and Roadmaps, a program of the University of Wisconsin Population Health Institute, that produces Narratives for Health; Narrative Initiative; and the Berkeley Media Studies Group, among others. However, the notion of building an infrastructure for narrative power has yet to be comprehensively detailed in literature within the domain of public health.

**Table 1:** Components of an Infrastructure for Building Narrative Power for Tobacco Control and Racism as a Public Health Crisis

		Tobacco Control Example: *Various Actors Engaged in Synchronous and Asynchronous Work Across Sectors That Built Narrative Power*	Racism as a Public Health Crisis Example: *Various Actors Have Begun to Mobilize to Build Narrative Power Around Organizing to Combat Racism*
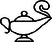	Identifying the goal	Initially public health and medicine sought to eliminate premature death and disease from smoking. Later, the public health goal was to protect the bystanders from secondhand smoke and ultimately protect them from the tobacco industry as an overall disease vector.	To articulate racism as a fundamental cause of health inequity and advance policy, system, and structural change to dismantle racism and improve health.
Infrastructure components:			
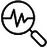	Research	Early studies demonstrated the health harms of smoking, but tobacco companies perpetrated false controversies that scared off funders. Researchers started to investigate Big Tobacco's false claims, and ultimately government and foundations invested public and private dollars in research that evolved from answering the demands of grassroots coalitions, to investigating how to effectively reduce tobacco use, and to more recent community‐led research as the foundation for policies that center equity.	Hundreds of studies have established not only that racism harms health but that racism is a fundamental cause of poor health and inequities. An early report from the Institute of Medicine (“Unequal Treatment”) was published in 2002. A follow‐up study by the National Academies of Sciences, Engineering, and Medicine, published in 2024, noted limited progress and named structural factors including racism as causes and recommended addressing structural racism as a way to eliminate health care inequities.
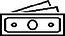	Resources	Early on, small organizations and grassroots groups scraped together resources for research. Only later, after they laid the foundation, did the national groups become engaged. Ultimately, resources for research and advocacy came from varied sources, as research institutions and funders saw the importance of reducing the harm of tobacco: federal dollars via the National Cancer Institute and the Office on Smoking or Health, excise taxes to support state and local action, state attorneys general negotiating the Master Settlement Agreement, private funds from foundations like the RWJF and donors through the American Heart Association, American Lung Association, American Cancer Society, and others.	Federal dollars via the NIH, CDC, and private funds from RWJF and others have been offered for research on structural racism. Schools of public health have developed centers and resources devoted to studying and addressing racism. See, for example, Berkeley Public Health's ARC 4 JSTC initiative, launched in 2020 to recognize its moral, ethical, and professional obligation to address racism based on the public health data.
		After secondhand smoke was established in a 1986 Surgeon General report, the organizations were more willing to get involved in an organized way. At the same time, tobacco companies were putting pressure on government and private funders to not do something about tobacco.	
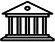	Litigation, legislation, and policy	Advocates brought lawsuits over local or state laws and put political pressure on legislatures and government agencies to pursue policies for clean indoor air, marketing restrictions, excise taxes, and limiting youth access. BIPOC communities were often at the forefront of calling for policy changes that targeted their specific needs but also created better health for everyone. In the 1990s, class action suits and state actions moved the discussion from individual smokers to the population harm.	More than 260 declarations at the state and local level have been made identifying racism as a public health crisis. The Institute for Healing Justice and Equity has model policies and guidance for how governments should respond.
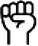	Organizing and advocacy	Building on the grassroots work in the 1970s and 1980s, organizers’ and advocates’ actions elevated an evolving narrative on tobacco control, from ASSIST in the 1990s (a partnership of the National Cancer Institute, 17 state health departments, and the American Cancer Society) that supported policy and media advocacy to the early 2000s with PATH (a partnership of The Praxis Project and RWJF) that supported local organizing focused on base‐building BIPOC organizations and cultivated leadership to the African American Tobacco Control Leadership Council whose work today is on demanding the FDA take action to eliminate menthol.	Upon the global public outcry—what some called a public reckoning—after the murder of George Floyd, many communities made declarations of racism as a public health crisis and some acted on them (see, e.g., endingracism.apha.org). However, other public agencies lacked the enduring partnerships needed with community organizations and grassroots groups.
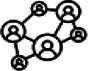	Networks	Early multiuser online bulletin boards like SCARCNet (eventually GLOBALNet) created an organized communication channel (fax machines and long‐distance calls were luxuries at the time) that presaged the way today's advocates can share information on social media. These early networks provided community and a common language, and regular meetings built trusting relationships and safe spaces to exchange ideas. Many networks, like the African American Tobacco Control Leadership Council and Americans for Nonsmokers Rights have focused on racial justice and on protecting workers.	The Collaborative for Anti‐Racism and Equity (CARE [herenow.org]) is one example, bringing together members from national organizations like APHA, ASTHO, NACCHO, BMSG, Big Cities Health Coalition, CEO Action for Racial Equity, ChangeLab Solutions, National Association of Chronic Disease Directors, Public Health Law Center, Salud America!, the Institute for Healing Justice & Equity, the Network for Public Health Law, and the Praxis Project.
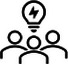	Training and support	Groups provided sustained policy and media advocacy training and support for public health advocates and researchers. *Columbia Journalism Review* prepared guides for journalists on reporting on tobacco. Trainings for advocates and organizers fostered shared framing around the harms from the industry and the role of government to rein it in. Advocates transferred their media advocacy skills to new policies as they won their battles. News coverage challenged industry.	CARE offers support, tools, and training to its members and others. The Race‐Class Narrative, among others, offers tools and training for talking more effectively about racism (but not specifically about racism as a public health crisis). Other trainings and presentations have been developed to help health professionals discuss racism as a determinant of health, including from public health leaders like Camera Jones.
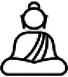	Reflection and evaluation	Policy change was a clear measure of success. However, there was more: those working in tobacco control created formal and informal mechanisms for reflection and evaluation. The Praxis Project's PATH created Learning Circles that brought tobacco control people together with others involved in health‐justice work to learn from each other. Over time, thousands participated, building long‐term narrative power.	Networks of leaders (such as CARE and other alliances) meet and reflect at conferences and convenings such as the APHA.
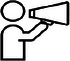	Communication	Researchers and advocates shifted the terms of debate through in‐depth analysis of the tobacco industry's narratives and then replaced those narratives about freedom of choice with strong, engaging narratives about health and freedom from harm. With media advocacy training, advocates created a common story for news and other media that shifted from a focus on individual behavior to fighting the corrupt tobacco industry. This was useful in engaging a broader base to act and in attracting investigative journalists to report on the tobacco industry. Artists defaced billboards and created counteradvertisements. Young people staged die‐ins to garner media attention. Hollywood produced movies vilifying the tobacco industry (and advocates are currently pressuring Hollywood to get smoking out of movies). African American communities organized to address particularly harmful tobacco industry practices, which catalyzed a greater focus on racial justice among advocates, media, and others across institutions and constituencies: medical and public health associations, unions, city councils and boards, schools, bars and restaurants, rodeos, health departments, base‐building and community‐based organizations, and congregations.	Many studies exist on problematic depictions of race and racism in news coverage and entertainment media, some connected to public health issues like violence but not necessarily to racism as a public health crisis.
			The Race‐Class Narrative, RaceForward, and others offer direct guidance about talking more effectively about racism (but not specifically about racism as a public health issue), and other academic and nonacademic researchers have developed research studies to understand the best approaches to communicating about racism. News coverage about racism as a public health crisis exploded during the summer of 2020. It tapered off after that but remains slightly higher than it was prior to 2020.
			One project that offers a powerful example of building sustained echo and reinforcing narratives across platforms is the work of Illuminative. Although not explicitly focused on public health, Illuminative uses a range of strategies, including narrative and culture change “to disrupt the invisibility of Native peoples, reeducate Americans, and mobilize public support for key Native issues.” They have supported the production of film and television series like Reservation Dogs, music projects, partnered with museums and other cultural institutions, and help conferences and symposia to raise issues of indigenous erasure and representation in media and academia.

Abbreviations: APHA, American Public Health Association; ARC 4 JSTC, Anti‐Racist Community for Justice and Social Transformative Change; ASSIST, American Stop Smoking Intervention Study; ASTHO, Association of State and Territorial Health Officials; BIPOC, Black, indigenous, and people of color; BMSG, Berkeley Media Studies Group; CARE, Collaborative for Anti‐Racism and Equity; CDC, Centers for Disease Control and Prevention; CEO, chief executive officer; FDA, Federal Drug Administration; NACCHO, National Association of County and City Health Officials; NIH, National Institutes of Health; PATH, Policy Advocacy on Tobacco and Health; RWJF, Robert Wood Johnson Foundation; SCARCNet, Smoking Control Advocacy Resource Center Network.

Adapted from the California Newsreel in collaboration with Higher Ground Change Strategies and Berkeley Media Studies Group.^35^

The processes and practices described in tobacco control occurred over decades and not necessarily all at once or in an orchestrated, top‐down manner. Prior to the Nonsmokers’ Rights Movement beginning in the early 1970s, tobacco was viewed as a medical problem. With the advent of activism around clean indoor air, the narrative shifted as the infrastructure was created by movement actors.

Narrative infrastructure to support movement building for health and racial equity is more than capacity building for actors making change. Building lasting narrative power requires interconnected support systems that foster networks of organizers, advocates, researchers, and media makers who can learn from one another and cement relationships and commitments to creating our healthy future. This infrastructure is not neutral. Across different disciplines, building narrative power requires uprooting dominant narratives and rooting health equity narratives so that they are reproduced by key institutions of meaning making and eventually become accepted as common sense. Column one in Table 1 identifies the key components of narrative infrastructure that are necessary to advance this goal.

Narrative infrastructure can include typical communications activities like training spokespeople and developing and disseminating messages but will go beyond those sorts of activities to seeding principled, visionary collaborations among communities most impacted by public health challenges as well as partnerships with public health practitioners, researchers, and community‐based organizations. Narrative infrastructure would support research and legal activities that are not typically considered communication per se. From that perspective, communication may be the most visible part of the infrastructure for building narrative power, but it is not the main part. A robust narrative infrastructure would help people craft more cohesive strategies to support intersecting work across issues like housing, health care, and jobs. This infrastructure would draw from partnerships that help build public health's capacity to ground work in a racial justice and system change framework; identify the biomedical, legal, social science, and other forms of research that help us determine which change strategies work; and provide mechanisms for evaluation and reflection.

An infrastructure for building narrative power would help public health practitioners connect the transactional tools and tactics that advocates use in their present advocacy, organizing and communications work to the longer term transformational change that will institutionalize health and racial equity. Importantly, multiple forms of power are required to achieve transformational change. The dimensions of power represented in this infrastructure are not mutually exclusive. We need various forms of narrative power working reciprocally and symbiotically as we organize individuals to act collectively in their interest to affect change, develop coalitions to organize organizations, conduct research to evaluate what works, advocate for policy change, and more across all the components of the infrastructure. Ultimately, narrative infrastructures support local, state, and national groups as part of the larger network of movement actors to bring together capacities for research, legal analysis, and policy; procure dollars to support and sustain the work; organize, advocate, and build local and regional networks; and support reflection, healing, and rejuvenation.

## Illuminating the Narrative Infrastructure in Tobacco Control

To make these concepts more concrete, we turn now to the public health and narrative success of tobacco control. The tobacco control movement built narrative power to drastically shift social norms and dominant narratives, first around smoking and eventually around tobacco itself. Although there are still issues to be addressed and smoking prevalence is still too high, we can be confident that our society will never go back to a state in which it is normal and encouraged to smoke in public places. The tobacco control movement wielded its power to transform many institutions, and establishing narrative power was an important part of that work.

Over the course of decades, public health advocates and journalists were able to wrest control of rhetoric about smoking away from the tobacco industry.^53^ As they fought for excise taxes, clean indoor air, and other policies, they transformed the dominant narrative from smoking as a personal choice to tobacco as a public health hazard. Indeed, once policies were implemented, a process of “policy feedback” occurred through which policies themselves shifted the public's beliefs.^54^ Building this kind of narrative power, alongside other forms of power, paved the road for systemic solutions like excise taxes, regulations to keep secondhand smoke out of public spaces, or changing the product itself (like eliminating menthol). This idea of *tobacco as a public health hazard* has been replicated, reproduced, and reified since the 1960s, and it is now the dominant narrative on tobacco.

In the case of tobacco control, advocates built a strong infrastructure over time to advance narrative power, enabling people across the country to share knowledge and bring antitobacco efforts to scale. In Table 1 (column two) we provide examples of various components of an infrastructure for building narrative power that emerged in the tobacco control movement (see also Appendix 2 for questions to consider in building these components).

Americans for Nonsmokers Rights was an early leader, supporting groups that created change at the local level before larger national groups came on board.^55^ This infrastructure was built in real time as different local and then state efforts gathered steam and learned from each other. Research on the health harms of smoking played a critically important role in the infrastructure. Ultimately, people across sectors began to see that it was reasonable to hold the industry accountable and to call on the government to act. They also reduced the cultural influence of tobacco by developing school curricula that taught kids the dangers of smoking, conducting media campaigns that warned of the dangers of tobacco but also called out industry, and even meeting regularly with filmmakers and screenwriters to advocate for limiting children and teens’ exposure to smoking in media and for more realistic portrayals of the harms of smoking in films made for adults.^56^, ^57^, ^58^, ^59^, ^60^

The result was a wholesale transformation of how, as a nation, we regard tobacco. After years of local grassroots efforts, foundations and government eventually provided support for researchers to evaluate promising policy solutions, advocates to bring those policies to life at the local level, and residents to engage their neighbors in forging healthier communities. Working and learning together, various configurations of these groups developed effective strategies for countering Big Tobacco's rhetoric and diminishing its influence in policymaking.^61^, ^62^, ^63^, ^64^

Transforming the narrative on tobacco took hard work, forward thinking, and robust support for research, advocacy, and communication. Change came through consistent effort over time despite serious lobbying and other activities by the tobacco industry to try to quash public health efforts. This is not to say that the movement has operated in lock step. It has certainly changed emphasis at different times, and different groups have focused on various policy initiatives—even some that have been considered contentious by fellow advocates including, currently, whether to treat vaping as a harm or harm reduction.^65^, ^66^ Although there has not always been agreement on the best next step, fundamentally, advocates have remained aligned on maintaining a lasting dominant narrative that tobacco is a health hazard and solutions require systemic changes.

## Building Narrative Infrastructure Around Racism as a Public Health Crisis

As noted earlier, the research is clear that racism undermines public health outcomes.^42^, ^43^, ^44^ A number of local health departments and national agencies including the Centers for Disease Control (in a declaration by the Centers for Disease Control and Prevention director, Rochelle Walensky, in 2021), the American Public Health Association, and the American Medical Association have declared racism a public health crisis, which helps to expand the narrative around health and racism.^67^ Scholars have argued, as we do, that narrative change is a necessary strategy to address racism.^68^ Yet, it would be naïve to imagine that the narrative infrastructure that was so important in the context of system and policy change surrounding tobacco would be as simple to apply to advancing the narrative that racism is a public health crisis and shifting a harmful narrative. First, racism is even more deeply embedded in US culture than tobacco use. There are entrenched narratives about race as biologically determined, racial inferiority and superiority, culture as causation for health outcomes, and racism as a natural and unsolvable behavior that is “baked into” human nature.^69^, ^70^, ^71^ These narratives are rooted and reinforced in all the institutions of meaning making including school, mass media (news, movies, gaming and other entertainment), and museums.

In 2020, the harmful impact of racism took center stage in the aftermath of the murder of George Floyd by a Minneapolis police officer. As tens of millions were in the streets fighting for some measure of justice, institutions were held to task regarding their efforts to address racism. For a moment, there was an international public conversation about racism and its devastating effects on communities. Some public health organizations moved from statements into actions, whereas other public agencies lacked the enduring partnerships needed with community organizations and grassroots groups.^72^ Advocates worked to win concrete changes that would help move resources toward advancing racial justice, and millions of dollars were pledged and invested in advocacy groups as a result. News coverage about racism as a public health crisis peaked that summer but waned after a few months because this narrative did not become rooted in public discourse or in any of the power structures that help us make meaning over time.^73^, ^74^ Similarly, although many organizations launched diversity, equity, and inclusion (DEI) initiatives in 2020, many were dismantled because they were also not successfully rooted in funding and organizational institutions and have been susceptible to shifting political winds. Still, these efforts could lay the groundwork for future racial justice movements.

Rooting the narrative about racism as a public health crisis will require public health advocates to develop more comprehensive narrative strategies than “piggybacking” on news events (like acts of violence against Black people) to capture public attention. Establishing narrative power in this context will require intentional and coordinated strategies for building the capacity of community organizations, public health advocates, local health departments, and other partners to organize and advance racial justice narratives to capture key institutions of meaning making so that these narratives are rooted and reproduced over time (see Table 1, column three). This process might look like sustained investments in advocacy networks, special funding opportunities created to continue to build the evidence base about interventions to advance racial equity, creative film projects, or listing racism as a contributing factor for specific diseases as a matter of protocol.^75^

Clearly, advocates will need to be creative given the plethora of successful attacks by the state against even talking about racism in the classroom and other public spaces. As early as 2022, some public health departments were forbidden to use the word “equity,” and these forces have been emboldened in 2025 by federal attacks on DEI.^76^, ^77^ Even a cursory examination of the highly resourced, organized, and savvy opposition to racial justice movements reveals the importance of narrative infrastructure both in terms of movement capacity and in developing supportive infrastructure rooted in structures that make meaning.^78^ Recent attacks on writers, books, courses, scientists, and institutions that teach, write, and conduct research around DEI, attacks on artists who express these values in their work, and news media attention to opponents of DEI and critical race theory (CRT) underscore how anti‐equity forces prioritize the capture of narrative infrastructure to advance their ideas and values.^79^ These forces have captured the narrative around race and racism through exposing and, thus, inoculating the public to messages that serve as counterarguments against equity and core public health values of social justice by fanning the flames of xenophobia with fear and angst.^38^, ^80^ This is not a debate about programs or finding the best way to rectify historic injustices. The coordinated attacks on affirmative action, CRT, and programs to institute DEI are strategies for expanding narrative territory by twisting the ideas to mean their opposite. Rather than rectifying past injustices or ensuring our institutions benefit from everyone's contributions, anti‐DEI forces are claiming that DEI is about exclusion.

To be sure, racism is among the most challenging issues to address because it is deeply interwoven throughout every aspect of American society—from individual thinking and belief to the most macrolevel of systems. There are centuries of narrative stitching all of it together into a deeply held overarching narrative. Even our understanding of whether taking on racism is “too political” for public health comes out of a dominant narrative that dictates what is political and what is apolitical. Changing these narratives requires a comprehensive, cohesive, and organized approach that prioritizes long‐term investment in the infrastructure and helps us build sustained and powerful movements to transform the systems and structures that shape our world and worldview.

## Conclusions

Our two examples, tobacco control and racism as a public health crisis, demonstrate the juxtaposition and nuance between the social determinants of health and the structural determinants of health.^10^ With tobacco control, the effective interventions were enacted within the social determinants of health. The transformation was about how people viewed responsibility for health problems related to tobacco, which public health advocates expanded to include government and industry but not necessarily how we view the role of government writ large. With racism as a public health crisis, effective interventions will need to be at level of the structural determinants of health, including the narratives about individualism, deservingness, and race. Changing these upstream social structures requires the power to “change decisions, agendas, and worldviews”^10^ and, as we have argued, the act of doing so requires developing a robust infrastructure for building narrative power.

To achieve health and racial equity in public health, we must expand our notion of communications to include a nuanced understanding of power, including the relationship between narrative power and the structural determinants of health. It will take significant investment of resources and coordinated, long‐term, cross‐sector effort. Public health practice and research often focus intently on single issues (e.g., smoking, nutrition, housing, etc.) to understand and address their nuance. However, public health as a discipline crosses issues because of its analysis of social and structural determinants of health and its attention to the root causes of morbidity and mortality that cross many issues at once and converge upstream. Therefore, whether individual public health practitioners and researchers focus on one or more issues at a time, the infrastructure for building narrative power must be activated within and across issues. Efforts to bring health to all policies is one tangible framework demonstrating the potential of working across issues and across sectors (although it also illuminates the challenges of doing so^81^).

Societal backlash to public health and to DEI represent huge challenges. At the same time, these countervailing pressures signify why timely attention to building an infrastructure for narrative power is crucial. An effective narrative change effort to address racism requires a restructuring and redistribution of work and resources in ways that break traditional silos, facilitate multisector, multidisciplinary collaboration, and cultivate broad community leadership and engagement. Fortunately, public health has a history of stepping up to some of the nation's most challenging issues. With intention and support, public health can build narrative power by rooting and reinforcing stories across the many institutions and cultural spaces that shape what we think, know, and believe. Public health will gain narrative power by changing or creating the institutions that reproduce our worldview not only in what our institutions say but also in what they do.

## Funding/Support

This research was supported, in part, by the following sources: The California Endowment (Dorfman, Tamber, Themba) and the Robert Wood Johnson Foundation (Dorfman, grant #81590; Gollust, grant #79754). The views expressed here do not necessarily reflect the views of The California Endowment or of the Robert Wood Johnson Foundation.

## Conflict of Interest Disclosures

The authors have no conflicts of interest to disclose.

## Appendix

 [Table milq70047-tbl-0003]

**Table A1:** Mapping Concepts of Narrative Infrastructure to Different Rubrics for Building Power

Narrative Infrastructure^a^		Power Prism^b^	Power Flower^c^	Three Faces of Power^d^
	Research	Research and data collection	Research and legal	
	Litigation, legislation, and policy	Campaign planning and decision‐maker advocacy	Research and legal, advocacy and policy	Legal advocacy
	Resources	Fundraising and development	Organizational development, infrastructure and funders	
	Organizing and advocacy	Grassroots and key contacts and decision‐maker advocacy	Organizing and base building	Organizing
	Networks	Coalition building and maintenance	Alliance and coalitions	Networks and deep alliances (beyond Alinsky)
	Training and support	Campaign planning	Leadership development	Leadership development
	Reflection	Campaign planning		
	Discourse and content analysis	Media advocacy	Communications, cultural, and narrative change	Countering the neoliberal and racist overarching narrative
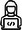
	New frames, stories, and messages	27‐9‐3 Elevator pitch and media advocacy	Communications, cultural, and narrative change	Countering the neoliberal and racist overarching narrative
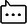
	Noise!	Media advocacy	Communications, cultural, and narrative change	Countering the neoliberal and racist overaching narrative
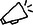
	Echo chamber	Media advocacy	Communications, cultural, and narrative change	Countering the neoliberal and racist overarching narrative
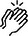

^a^ Adapted from California Newsreel, in collaboration with Higher Ground Change Strategies and Berkeley Media Studies Group.^35^

^b^ Power Prism https://mypowerpeople.com/about‐power‐prism

^c^ Health and Justice for All Power‐Building Landscape: A Preliminary Assessment. USC Program for Environmental and Regional Equity. (October 22, 2018), available from https://dornsife.usc.edu/eri/wp‐content/uploads/sites/41/2023/01/2018_TCE_PLA_PERE.pdf and cite is Health.

^d^ The Three Faces of Power. Available at: https://grassrootspowerproject.org/analysis/the‐three‐faces‐of‐power/

**Table A2 milq70047-tbl-0003:** Questions to Consider for Building Power Across Narrative Infrastructure Elements

Component		Questions to Consider for Building Power
	Research	What causes the problem? What will fix it? What data do we have and what data do we need—and what are considered “data” to begin with? How are the data of lived experience brought forth? Are the people most harmed involved in shaping the research? What's been done before? What are the bold ideas others are testing? Will they work here?
	Litigation, legislation, and policy	What mechanisms should we use for structural change? Legislation? Litigation? Rulemaking? Local policy? Institutional policy? How are those who have been most harmed centered in moving forward on litigation, legislation, and policy?
	Resources	Where will the resources come from and where they will go? Where have they gone previously? Who makes decisions about resources? Who will advocate for more and who will write the checks—or force checks to be written?
	Organizing and advocacy	Who is most impacted? How does the organizing support their leadership? What changes specific to the issue will contribute to our collective vision for a more just world? Who conducts the power analysis? Who makes decisions about what tactics and approaches will be a part of the advocacy? Who decides which stories will be centered in advocacy? How can the work on this specific issue build longer term narrative power?
	Networks	Who comes together, and how? How does our issue connect to others? Where can we join forces or reinforce one another? How can we ensure our networks center those most harmed and are guided by a focus on racial and health equity? What can we gain in both organizing and narrative power when we work in this way?
	Training and support	Who gets training and practice? Who is able to share stories as someone who is harmed by current policies? Who is portrayed as an expert? Are those who are most harmed being portrayed as data, health, policy, or other experts? Are they being supported in highly visible, high stakes situations? Are we providing each other the solace and comfort we all need as we go through loss, pain, and grief as we do the work? Even if our trainings and support are focused on a near‐term issue, how are they building long‐term narrative power?
	Reflection	What constitutes progress? Who determines what is working and what needs to change? What parts of our work led to transformational narrative change that fundamentally shifts power to change the oft‐repeated, deeply ingrained stories that we believe about this issue? What parts were more transactional—maybe they contributed to some wins, but they didn't shift how people think about the issue? How could this shift in the future?
	Discourse and content analysis	What is the dominant story about our issue? How can we combat the flaws in the dominant narrative? What narrative do we want to offer as a counter to the dominant narrative?
	New frames, stories, and messages	What stories will communicate our values and vision for a just world? What is the best way to counter opposition? What is the best way to expand our base of engaged communities and advocates who will amplify the narrative and push for change? What messengers will convey these messages?
	Noise!	Where and when should we create news? Purchase ads? Submit opinion pieces? Write plays, novels, movies? Produce music? Art? Editorial cartoons? Textbooks? Perform comedy? Use direct actions?
	Echo chamber	How do we connect our story with others? Who hasn't heard the story that should? Whose voices need to be added or elevated?

Source: California Newsreel, in collaboration with Higher Ground Change Strategies and Berkeley Media Studies Group.^35^
